# Effects of fibrillin mutations on the behavior of heart muscle cells in Marfan syndrome

**DOI:** 10.1038/s41598-020-73802-w

**Published:** 2020-10-07

**Authors:** Jeffrey Aalders, Laurens Léger, Louis Van der Meeren, Natasja Van den Vreken, Andre G. Skirtach, Sanjay Sinha, Julie De Backer, Jolanda van Hengel

**Affiliations:** 1 Medical Cell Biology Research Group, Department of Human Structure and Repair, Faculty of Medicine and Health Sciences, Ghent University, Corneel Heymanslaan 10, Building B, Entrance 36, 9000 Ghent, Belgium; 2 Department of Biotechnology, Faculty of Bioscience Engineering, Ghent University, Ghent, Belgium; 3 Wellcome-MRC Cambridge Stem Cell Institute, University of Cambridge, Cambridge, UK; 4 Center for Medical Genetics, Ghent University Hospital, Ghent, Belgium

**Keywords:** Cardiology, Cardiovascular diseases, Biological models, Cardiovascular models

## Abstract

Marfan syndrome (MFS) is a systemic disorder of connective tissue caused by pathogenic variants in the fibrillin-1 (*FBN1*) gene. Myocardial dysfunction has been demonstrated in MFS patients and mouse models, but little is known about the intrinsic effect on the cardiomyocytes (CMs). In this study, both induced pluripotent stem cells derived from a MFS-patient and the line with the corrected *FBN1* mutation were differentiated to CMs. Several functional analyses are performed on this model to study MFS related cardiomyopathy. Atomic force microscopy revealed that MFS CMs are stiffer compared to corrected CMs. The contraction amplitude of MFS CMs is decreased compared to corrected CMs. Under normal culture conditions, MFS CMs show a lower beat-to-beat variability compared to corrected CMs using multi electrode array. Isoproterenol-induced stress or cyclic strain demonstrates lack of support from the matrix in MFS CMs. This study reports the first cardiac cell culture model for MFS, revealing abnormalities in the behavior of MFS CMs that are related to matrix defects. Based on these results, we postulate that impaired support from the extracellular environment plays a key role in the improper functioning of CMs in MFS.

## Introduction

Marfan syndrome (MFS) is a rare systemic disorder of the connective tissue with an estimated prevalence of 1:5000–1:10,000^1^. Typically, multiple organ systems are affected in MFS patients, with manifestations in the skeletal, ocular, integumentary, respiratory and cardiovascular system^2^. Aortic complications due to progressive aortic dilation leading to potential aneurysm, dissection, and rupture are the main cause of morbidity and mortality in patients with MFS.

Pathogenic variants in the *FBN1* gene, coding for fibrillin-1, are causative for MFS^3^. Fibrillin-1 is a major component of the microfibrils that are important in the extracellular matrix (ECM) including the ECM of elastic tissues such as the aorta^4^. The localization of fibrillin-1 in the heart also suggests a role for fibrillin-1 in myocardial tissue^5^. Due to pathogenic variants in *FBN1,* elastic fiber composition is suboptimal and compensated by excessive collagen and proteoglycan deposition, which leads to increased stiffness and progressive weakening of the ECM^6^.

In addition to structural and mechanical support, fibrillin-1 also exhibits regulatory activities in growth factor signaling, ECM formation, cell behavior and the immune response^7^. Microfibrils normally act as docking sites for latent TGF-ß complexes, however, pathogenic variants in *FBN1* result in release and activation of the normally bound TGF-ß^8^. While increased TGF-ß signaling is a hallmark of MFS, uncertainty remains about the molecular mechanisms and disease progression^9,10^.

While aortic complications are still the leading cause of MFS-related mortality, advances in medical and surgical management have improved life expectancy^11^. Due to this increased life expectancy, other clinical manifestations have arisen, among which is myocardial involvement^12^. Myocardial dysfunction secondary to significant valvular disease is a well-known cardiovascular complication in MFS^13–15^. However, several independent studies have provided evidence for MFS-related cardiomyopathy unrelated to valvular disease, leading to the term Marfan cardiomyopathy^12,16–18^.

While *FBN1* appears causative for MFS cardiomyopathy, these studies also warrant the necessity for a better understanding of the underlying mechanisms. An approach to study MFS cardiomyopathy could be by collecting CMs from MFS patients during surgery, transplantation or biopsy, but this is a rather invasive and limiting method to study the disease. In vivo mouse models for MFS with fibrillin-1 deficiency have led to an increased understanding of MFS. Abnormal mechanosignaling by CMs has been observed in mouse models for MFS that can lead to dilated cardiomyopathy, thus implying an intrinsic cardiomyopathy^14^. However, the mouse model has some limitations. For instance the beat rate of the mouse heart differs from that of the human heart^19^.

An alternative approach to in vivo human studies and animal studies is through the generation of stem cell derived CMs. Somatic cells of MFS patients could be reprogrammed to human pluripotent stem cells (hiPSCs)^20^. An unlimited supply of CMs can be differentiated from hiPSCs with good potential for an in vitro model that resembles the human cardiac tissue and accurately recapitulates the human cardiac pathophysiology^21^. This approach has led to improved understanding of various other genetic cardiomyopathies^22–24^. However, to the best of our knowledge, no in vitro cardiac model has been described for MFS. An in vitro cell model offers the possibility to analyze specific cell types outside their complex biological context and excludes in vivo masking factors such as the effect of specific medical treatment.

The hiPSC strategy has been employed previously to establish a vascular model of MFS, which investigated disease mechanisms in smooth muscle cells^25^. This current study reports the functional characterization of the in vitro MFS cardiac model that was derived by differentiating hiPSCs to CMs. The established in vitro cardiac model for MFS was studied by means of multi electrode array (MEA), cyclic strain imparted with the Flexcell, atomic force microscopy (AFM) and video analysis, revealing abnormalities in the behavior of MFS CMs.

## Material and methods

All products used are purchased from Life Technologies unless mentioned otherwise.

### Culture of hiPSCs

hiPSCs MFS and hiPSCs corrected lines were obtained via prof. S. Sinha (University of Cambridge) which were previously generated and characterized by his group^25^. These are isogenic lines; MFS harbors a *FBN1* mutation in exon 30 (c.3725G > A), while in the corrected hiPSCs this mutation was repaired using CRISPR-Cas9. For CRISPR-Cas9, a donor plasmid with intronic homology arms 1,063 bp upstream and 970 bp downstream of exon 30 was used for specific homology direct repair for the mutant allele CYS1242TYR. These cell lines were originally cultured on mitomycin-C-treated MEFs in hiPSC medium and maintained at 37 °C, 5% CO_2_ and 5% O_2_. hiPSC medium contained: DMEM/F12 (Cat No. 31330038), 20% Knock-out serum replacement (Cat No. 10828028), 2 mM l-glutamine (Cat No. 25030081), 1% Non-essential amino acids (Cat No. 11140-050), 0.1 mM β-mercapthoethanol (Cat No. 31350010), 100 u/ml Penicillin and 100 µg/ml Streptomycin ((P/S) Cat No. 15140-122) and further supplemented with 4 ng/ml bFGF-2 (PeproTech, Cat No. 100-18B-10UG). Cultures were adapted to feeder-free conditions using Geltrex coating (Cat No. A1413302) and cultured in Essential 8 medium (Cat No. A1517001) supplemented with P/S with a seeding density of 2.1 × 10^4^ cells/cm^2^ ^26^. The H9 human embryonic stem cell (hESC) line (WA09, WiCell, feeder free cultures were obtained via prof. C. Verfaillie, KULeuven, Belgium) was cultured feeder-free in Essential 8 medium under the same conditions as the MFS and corrected lines and was used as control.

### Differentiation to cardiomyocytes

For the derivation of CMs from hiPSCs and H9 stem cells, the PSC Cardiomyocyte Differentiation Kit (Cat No. A2921201) was used, the manufacturer’s protocol was slightly modified. In short, hiPSCs were transferred in clumps using Versene/EDTA (Lonza, Cat No. 17-711E) to a Geltrex coated 12-well with a seeding density of 5.7 × 10^4^ cells/cm^2^. After 2 days, the Essential 8 medium was supplemented with 1:100 Geltrex to create a sandwich overlay. After 3 days (at 70–80% confluence) the cells were washed with PBS and the differentiation was induced according to the protocol of the PSC Cardiomyocyte Differentiation Kit. From day 4 of differentiation the cells were kept in maintenance medium and medium was refreshed every 48 h.

### Passaging of cardiomyocytes

In vitro derived CMs were passaged for subsequent experiments using TrypLE select enzyme (Cat No. 12563011). CM cell cultures were washed with PBS and subsequently incubated for 5 min with TrypLE at 37 °C. Cells were dissociated by pipetting gently up and down and TrypLE was inactivated by diluting in maintenance medium. Cell suspension was centrifuged for 5 min at 200×*g*, and pellet was dissolved in maintenance medium supplemented with 1:100 RevitaCell (Cat No. A2644501) and 1:100 Geltrex for 2 days with a seeding density of 2 × 10^5^ cells/cm^2^. Maintenance medium was then refreshed every 48 h.

### Immunohistochemical analysis of stem cells and cardiomyocytes

hiPSCs and CMs were fixed for 20 min with 4% paraformaldehyde at RT. hiPSCs were permeabilized for 30 min with 0.1% Triton ×-100 diluted in phosphate buffered saline (PBS). Subsequent incubation with blocking solution consisting of 5% Goat serum (Cat No. 16210-064) in PBS was done for 30 min. The cells were incubated overnight at 4 °C with primary antibodies diluted in PBS containing 0.05% Tween20 and 1% bovine serum albumin (BSA). The used antibodies are listed in Supplementary Table 1. The next day, the cells were incubated for 30 min at RT with secondary antibodies diluted in PBS containing 0.05% Tween20 and 1% BSA and subsequently incubated for 10 min with 0.1% Hoechst solution (Cat No. H3570). The immunostaining of CMs was performed as previously described with the exception that the primary antibody was incubated overnight at 4°C^27^. Microscopy images were made using the EVOS FL Imaging System or the ZEISS LSM900 confocal microscope.

### Gene expression analysis of cardiomyocytes

RNA extraction was performed from cell cultures at day 15 and 24 after start of differentiation to CMs in accordance with the manufacturer’s protocols using GeneElute Mammalian Total RNA Miniprep kit (Sigma-Aldrich, Cat No. RTN70) including on-column DNA digestion. RNA was converted to cDNA using the SuperScript III First-Strand Synthesis SuperMix-kit (Invitrogen, Cat No. 18080400). qPCR was performed using the Platinum SYBR Green qPCR SuperMix-UDG-kit (Cat No. 11733038) on a CFX96 Touch Real-Time PCR Detection System (Bio-Rad) using the following PCR protocol: 2 min 50 °C, 2 min 95 °C followed by 40 cycles of 20 s 95 °C and 45 s 60 °C on 10 ng cDNA per 20 µl reaction. Primers were used at a final concentration of 200 nM (Supplementary Table 2). Analysis of qPCR results was performed using CFX Manager-software (Bio-Rad).

### Atomic force microscopy

All AFM measurements were executed with the bio-AFM: Nanowizard 4 (JPK instruments). For these measurements, the DNP-S10 (Bruker) chip was used. On this chip, a V-shaped cantilever B was chosen, which has a nominal spring constant of 0.06 N/m and a nominal radius of curvature of 10 nm. High resolution images were made using the special Quantitative imaging (QI) mode, which allowed the acquisition of the mechanical properties of each pixel upon scanning. Large scale force maps were measured in the contact mode. All the measurements were executed with setpoints ranging from 2 to 5 nN. The AFM instrument was mounted on top of an inverted microscope (Zeiss) facilitating the localization of tissues. Areas of interest were selected based on morphology to ensure that representative parts of the culture were assessed. Furthermore, an in-house built incubator was mounted around the AFM, providing a 37 °C and 5% CO_2_ environment, permitting prolonged measurements without affecting cells.

The software used to acquire and process the AFM data was provided by the instrument manufacturer: JPKSPM version 6.1.165 (JPK BioAFM, Bruker, https://www.jpk.com/). Based on force curves, the Young’s modulus was calculated using the Hertz/Sneddon model adjusted for parabolic indenters^28,29^. After Young’s modulus calculation, the elasticity data was further normalized and filtered in MATLAB version R2019b Update 6. Statistical data analysis was performed in R-studio (version 3.5.0) using the Welch-two-sample T-test.

CMs grow as a layer on top of a basal layer, and showed as a distinct population in the AFM measurements. 20-day-old CMs were passaged to 35 mm plastic petri dishes and were analyzed after a 5 day recovery period. The beating of CMs was stopped directly before AFM measurements using a 40 mM KCl solution. In order to obtain mechanical information of this heart cell population, the Young’s modulus of the top 25% highest points in the QI-maps was compared to the overall average Young’s modulus of each cell line. To acquire this data, points obtained in the QI-force-height maps were pooled together for each cell line. From this pool, the average Young’s modulus was determined. Furthermore, the maximum height was determined and consequently the highest 25% was selected. Using the corresponding Young’s modulus of this selected highest 25%, the average Young’s modulus was recalculated and compared to the previous average calculated on the whole population.

### Video analysis using Musclemotion software

Musclemotion software was used to investigate difference in contraction between the corrected and MFS CMs^30^. Short video clips of 30 frames/s were made from beating corrected and MFS CMs for 60 s for three independent cell cultures per line. The algorithm used in this software compares the pixel intensity of each frame with a reference frame. The difference measured between each pixel in the reference frame and the pixel in the frame of interest, is given as output in absolute numbers. This number represents the change of displacement in time, and correlates with the contraction amplitude.

### Multi electrode array

Multi electrode array measurements were performed with a 6-well MEA (Multi Channel Systems, 60-6wellMEA200/30iR-Ti-rcr) on a USB-MEA-128 system with a MEA1060BC amplifier (Multi Channel Systems). Electrodes of each 6-well were coated with a 10 µl drop of 25 µg/ml fibronectin (PromoCell, Cat No. C-43060) for 1 h at 37 °C. Approximately 30.000 CMs (15–20 days old) were seeded onto those drops. After a recovery period of 5 days, baseline recordings measuring the extracellular electrograms of spontaneously contracting CMs were made for 180 s using the Cardio2D software (Multi Channel Systems). The temperature was kept at 37 °C using a heating element and a temperature controller. One electrode with the best signal was selected per well for subsequent analysis. Automatic beat detection was used in the Multi-well analyzer software (Multi Channel Systems) to derive RR intervals. The RR interval is the time between two consecutive membrane depolarizations, indicating the beat-to-beat duration. SDNN was calculated as the standard deviation of all the RR intervals. The SDSD was calculated as the standard deviation of the successive RR interval differences. Coefficient of variance is obtained by dividing the SDNN by the average RR interval.

### Isoproterenol treatment

Isoproterenol (ISO, Sigma-Aldrich, Cat No. I6504) was diluted to a 3 mM stock in distilled water and filtered with a 0.2 μm filter. Stock solution was freshly diluted in maintenance medium and only a maximum of 5 μl compound was added, minimizing medium changing effects. CMs were stimulated with increasing concentration (5 nM, 10 nM, 25 nM, 50 nM and 100 nM) of ISO. For each dose, extracellular electrograms were measured for 60–300 s at a sampling rate of 10,000 Hz.

For the chronic treatment, the medium of CMs was refreshed daily with maintenance medium containing 1 µM ISO, starting at day 17 for a period of 7 days.

### Flexcell

Flexcell FX-5000 Tension system (Flexcell International Corporation) was used to apply mechanical forces on the in vitro cell model. Bioflex 6-well plates were coated with drops (20 µl) of 25 µg/ml fibronectin for 1 h at 37 °C. CMs were seeded onto those drops at a density of ∼ 30.000 CMs/drop. CMs were stretched in the Flexcell FX-5000 tension system (sinus) with 10% stretch at 1.0 Hz for 6 h and 40 h. As a control, both corrected and MFS CMs were cultured on the Flexcell membrane without stretching.

### Statistical analysis

Statistical analysis for MEA data, gene expression levels and contraction amplitude derived from the video clips and Spearman’s rank correlation was performed using SPSS statistics version 23.0 for Windows (IBM Corp., Armonk, NY). Means of normalized expression were compared using unpaired t-test and reported as two tailed *p* value for comparison of two independent means with significance level of 0.05.

### Ethics statement

Experiments with hiPSCs were approved by the local ethical committee of Ghent University Hospital (EC UZG 2017/0855).

## Results

In this study, an in vitro cardiac model for MFS is established and investigated. CM cell cultures derived from hiPSCs carrying a pathogenic variant in *FBN1* are compared with CM cell cultures derived from the isogenic hiPSCs, where the pathogenic variant is CRISPR-Cas9 corrected^25^. The cell cultures are respectively named MFS and corrected. Furthermore, the hESC H9 line is used in MEA experiments as an additional control.

### Cell culture and cardiac differentiation potential not affected by FBN1 variant

**Figure 1:**
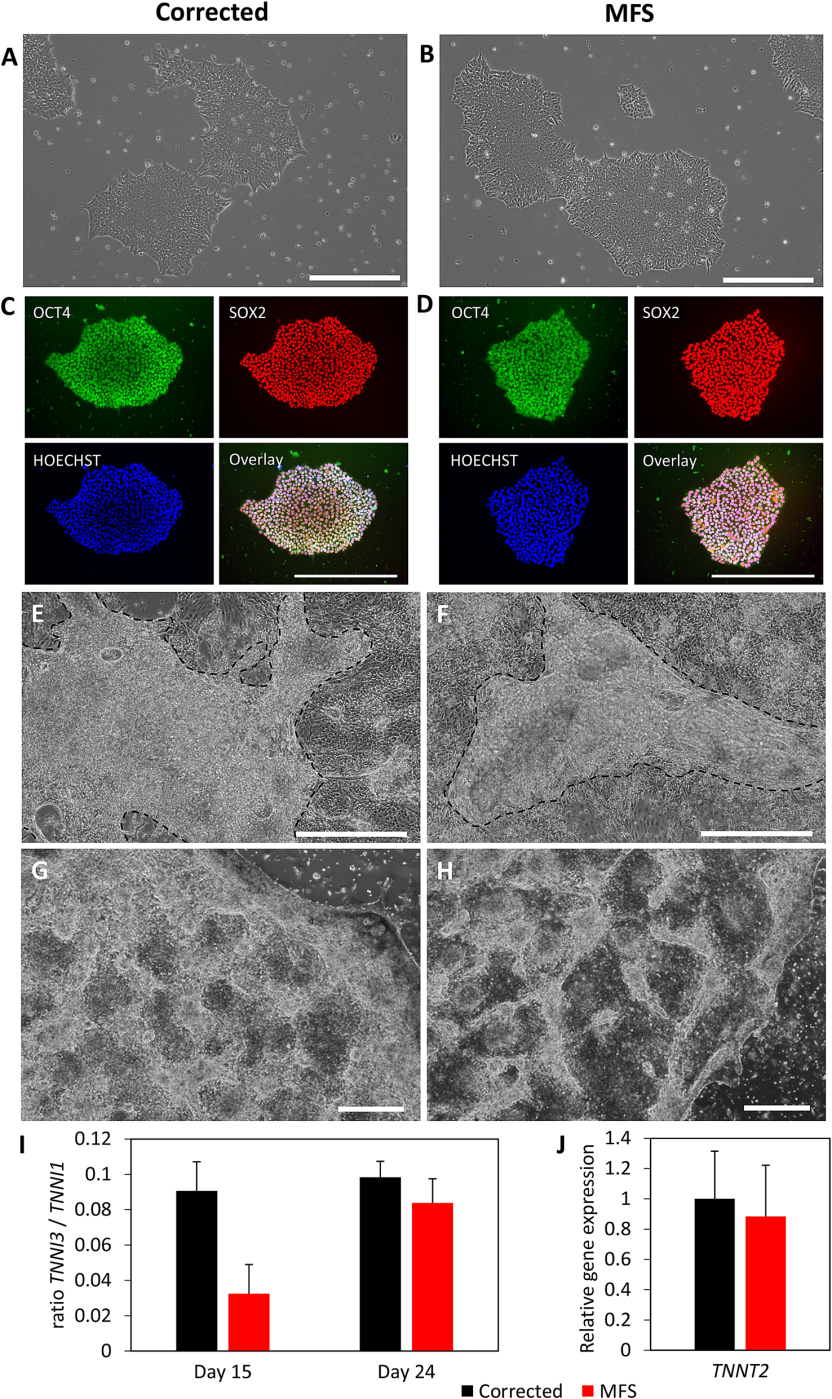
Phase contrast or immunofluorescent pictures from cell cultures of corrected (**A**,**C**,**E**,**G**) and MFS (**B**,**D**,**F**,**H**). hiPSC cultures have similar growth characteristics (**A**,**B**) and show expression of pluripotency markers OCT4 (green) and SOX2 (red) and HOECHST (blue) (**C**,**D**). Both CMs from corrected and MFS are growing as a layer on top of a basal layer of cells (**E**,**F**). The area of CMs in (**E**) and (**F**) is highlighted by the dashed line. Passaged CMs show better recovery in corrected (**G**) compared to MFS CMs (**H**) as indicated by the more brighter areas in corrected CMs. Scalebars indicate 500 µm. Graph (**I**) shows the ratio between *TNNI3* and *TNNI1* as a ratiometric marker for maturation of the CMs at day 15 and day 24, n = 3. Graph (**J**) represents the relative gene expression for *TNNT2* in the cardiac model at day 24 for corrected (black) and MFS (red), n = 6.

The hiPSCs cultures of MFS and corrected (Fig. 1A-B) show similar growth characteristics. Passaging of the cultures was done every 3–4 days at a confluency of approximately 80% with the same passaging ratio (1:4). Fluorescent intensities of OCT4 (2.13 vs 2.05), SOX2 (2.08 vs 1.53) and NANOG (1.40 vs 1.53) relative to HOECHST for the corrected and MFS hiPSCs respectively, show that both cell cultures are pluripotent (Fig. 1C,D and suppl. Fig. 1). Directed cardiomyocyte differentiation of the hiPSCs leads to beating CMs after 8–12 days after the start of differentiation for both MFS and corrected hiPSCs (Fig. 1E,F). In this culture system, the derived CMs grow as a contracting layer on top of a basal layer that was formed during the cardiac differentiation. It is presumed that the non-contracting basal layer exist predominantly of fibroblasts. The recovery of CMs after passaging is observed to be relatively lower for MFS CMs compared to corrected CMs as showed by smaller areas of contraction (Fig. 1G,H). CMs are passaged only once for subsequent experiments, taking into account this relative lower recovery rate of MFS CMs to have similar culture composition in these analyzes. The maturation status of the CMs was determined using the relative gene expression of *TNNI1* and *TNNI3,* showing a relatively lower maturation status for MFS CMs compared to corrected CMs at day 15 (Fig. 1I), although not statistically significant (*p* = 0.067). After 24 days of cardiac differentiation, the maturation status of MFS CMs increased, although not significantly (*p* = 0.074) and is similar to the corrected CMs. Moreover, no significant difference in *TNNT2* gene expression 24 days after cardiac differentiation is observed (Fig. 1J), indicating that the differentiation potential of the hiPSCs is not affected by the *FBN1* variant.

### Defective fibrillin in the MFS cardiomyocyte cell culture confirms ECM defect

**Figure 2:**
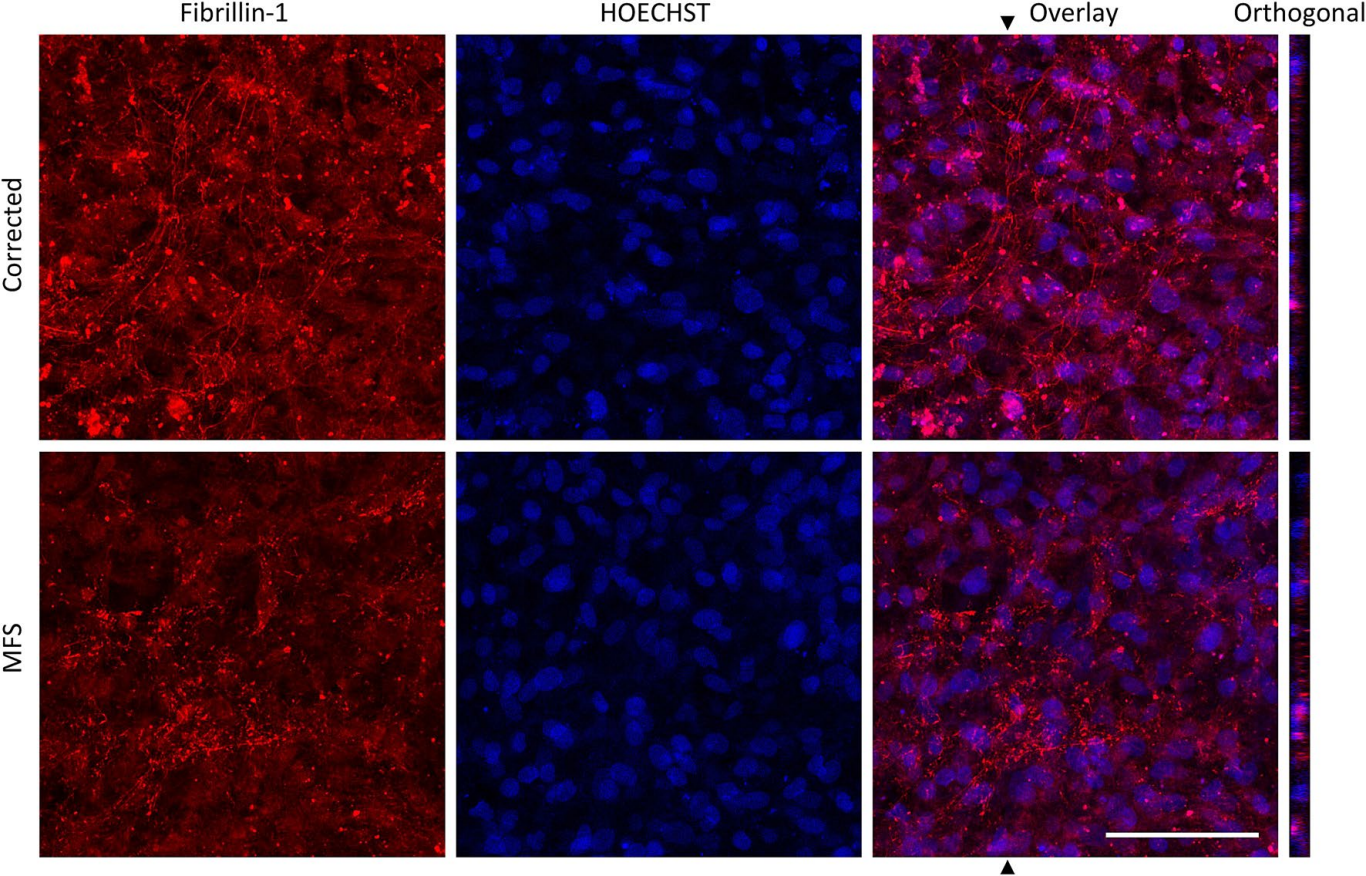
Immunohistochemical staining for fibrillin-1 on CM cell culture for corrected and MFS. Fibrillin-1 in red and nuclei stained with HOECHST in blue after 25 days after the start of cardiac differentiation. Fibrillin-1 microfibrils or punctate assemblies are observed in corrected or MFS cultures, respectively. The average fluorescent intensity of fibrillin-1 in this image is respectively 26.65 and 19.51 for corrected and MFS cell culture. Orthogonal view shows the z-section of the area, indicated with the arrow in the overlay image. Bar indicates 100 µm.

Immunofluorescent staining for fibrillin-1 after 25 days of CM cell culture shows that the fibrillin-1 fibers are scarcely present as punctuated assemblies in the MFS cell culture and abundantly present in the corrected (Fig. 2). This demonstrates the expected dysfunctional fibrillin-1 in the matrix of the MFS cell culture.

### Atomic force microscopy shows that cardiomyocytes in the MFS in vitro model are stiffer

**Figure 3:**
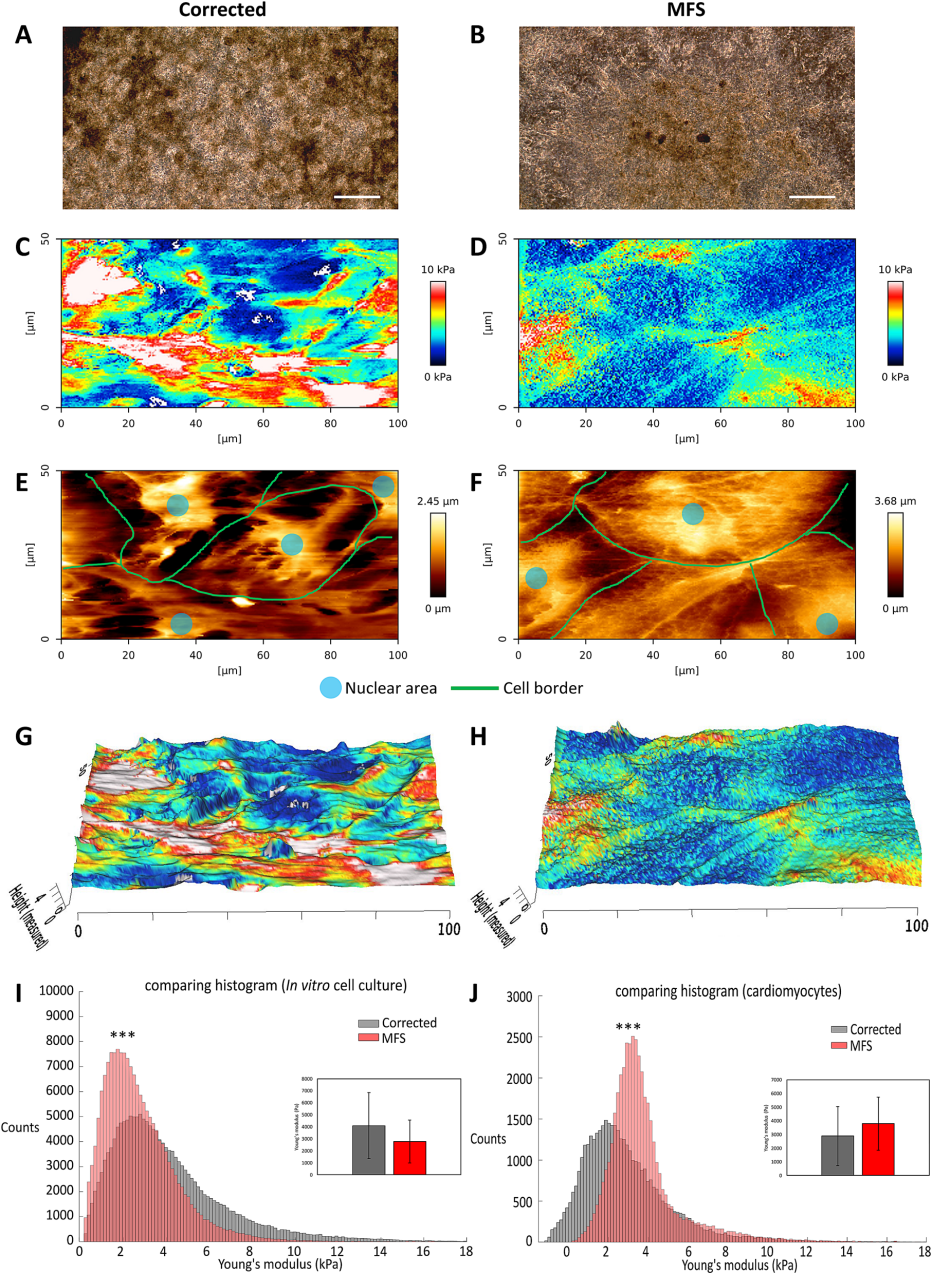
Atomic Force Microscopy of 25-day-old cardiomyocytes: phase-contrast images of corrected (**A**) and MFS (**B**) cell cultures the mechanical stiffness mapping for corrected (**C**) and MFS (**D**) and the topography mapping of the (height) for corrected (**E**) and MFS (**F**) (colours: red is stiff, blue is more elastic) for corrected (**C**) and MFS (**D**) cells. Annotation of cell border (green line) and nuclear area (blue) in (**E**) and (**F**). Mechanical stiffness mapping is overlaid with topography mapping for corrected (**G**) and MFS (**H**). Dimensions are indicated in µm. AFM was performed on three independent cultures for each cell line. Large scale measurements of the complete CM cell cultures show a higher average Young’s modulus for the corrected (black) cell culture in comparison to MFS (red) (*p* < 0.0001) (**I**). The CMs from MFS show a higher Young’s modulus compared to corrected CMs when only the 25% highest areas, composed of CMs, were measured (*p* < 0.0001) (**J**). The bars in (**I**) and (**J**) indicate the standard deviation. The level of significance is indicated by asterisks: *p* values less than 0.001 are indicated with three asterisks. Bar in panel (**A**) and (**B**) indicates 500 µm.

In the current study, an advanced quantitative imaging (QI) mode is employed on corrected and MFS cell cultures of (Fig. 3A,B), allowing simultaneous profiling of the surface topography and mapping of mechanical stiffness. Similarly as demonstrated in Fig. 1E,F, the recovery of corrected CMs is better than in MFS CMs, but both cultures show a beating layer of cardiomyocytes. Resulting QI images show a map of force (Fig. 3C,D) and height (Fig. 3E,F) of the 25 day old CMs cell cultures for corrected and MFS. Figure 3G,H project the stiffness, measured as the Young’s modulus, on top of the height of the cells. The CMs in this in vitro model are growing as a layer on top of a basal layer of cells as shown in Fig. 1E,F. The highest areas are shown as a distinct population in the AFM (highest 25%). In the in vitro MFS model the highest areas are the stiffest, while the opposite is observed in the corrected cell line, where the stiffest areas are located at the lower areas (Fig. 3G,H). The AFM images show that on average the corrected cell culture system is stiffer than the MFS cell culture system (Fig. 3I) (*p* value < 0.0001). These measurements are performed on the complete CM cell culture, including the cells in the basal layer. However, AFM measurements on the upper layer of CMs, show that the MFS CMs are significantly stiffer than the corrected CMs (Fig. 3J) (*p* value < 0.0001). The stiffness of the MFS CMs compared to the complete MFS cell culture where significantly stiffer (*p* value < 0.0001) and the corrected CMs were significantly less stiff compared to the complete corrected culture (*p* value < 0.0001).

### Video analysis using Musclemotion

**Figure 4:**
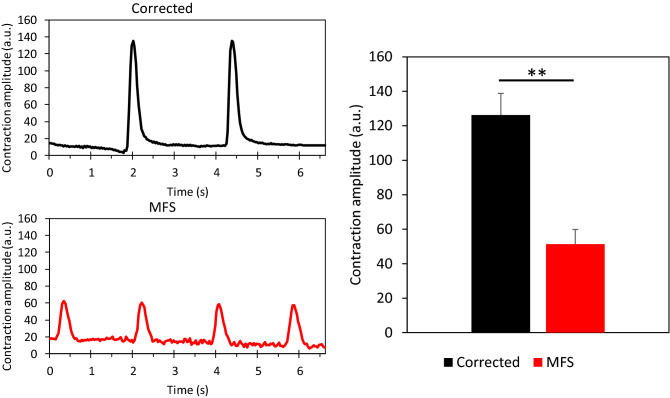
Video analysis of the beating of cardiomyocytes using Musclemotion was performed on corrected (n = 3, black) and MFS cell cultures (n = 3, red). The representative plots show differences in contraction amplitude. The bar graph shows the average contraction amplitude of three independent experiments per cell line and shows a significant difference (*p* = 0.0078), error bars represent the standard error in the bar graph. The level of significance is indicated by asterisks: *p* values less than 0.01 indicated with two asterisks.

In order to assess if the dysfunctional fibrillin in the ECM of the MFS CM cell culture impaired contraction, videoclips are analyzed using the Musclemotion software (suppl. video 1 and suppl. video 2). The 25-day-old MFS CMs have a shorter beat-to-beat interval compared to corrected CMs (Fig. 4). The corrected CMs have a significantly higher contraction amplitude compared to the MFS CMs (p = 0.0078), indicating that the corrected in vitro culture system exerts more relative displacement compared to the MFS.

### MEA results show distinct differences between MFS and corrected cardiomyocytes

**Figure 5:**
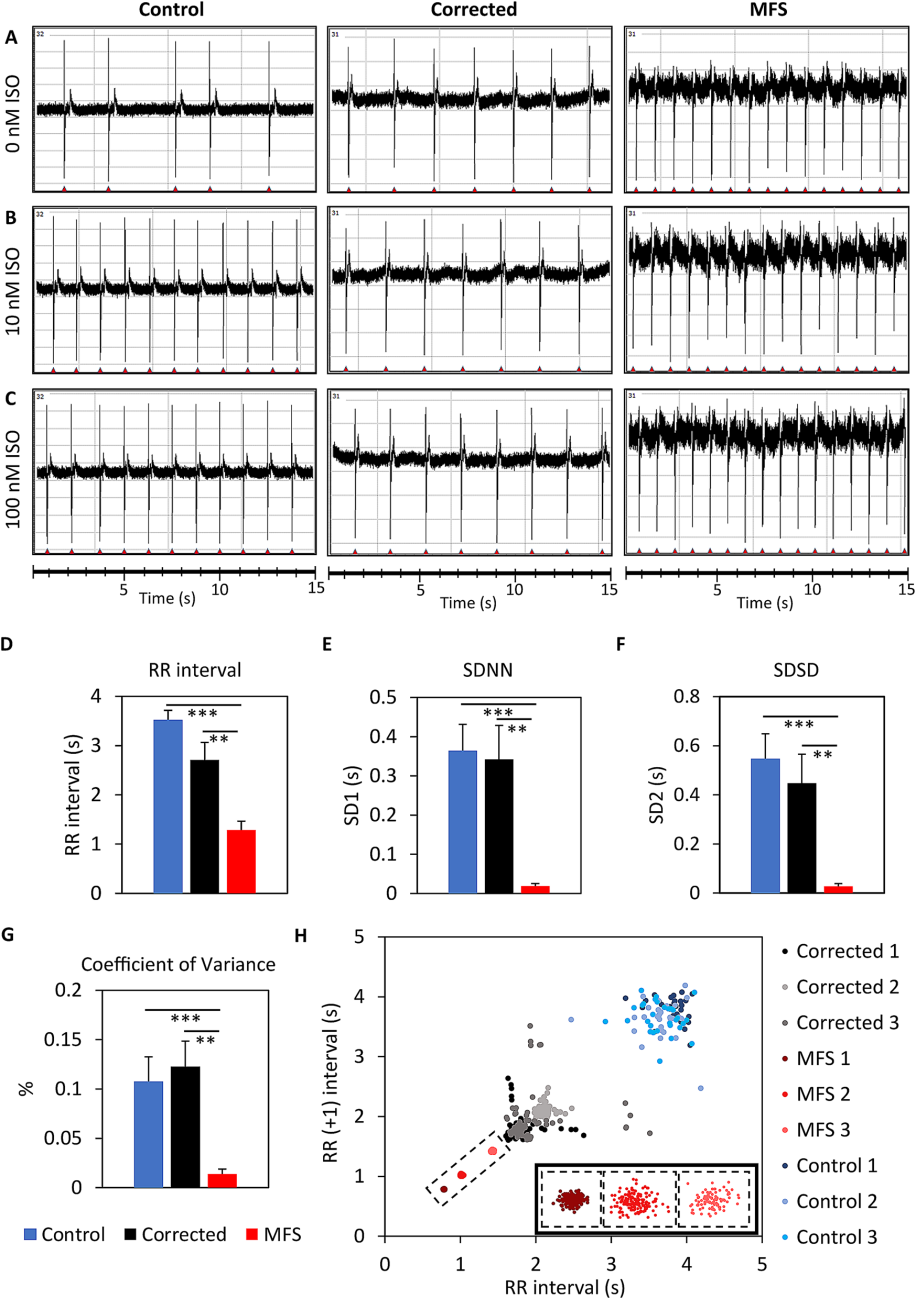
MEA measurements of the extracellular field potentials for the control line (H9), the corrected CMs and the MFS CMs after 20–25 days after start of cardiac differentiation. (**A**) The baseline measurements without the addition of isoproterenol (ISO). The extracellular field potentials are shown for 10 nM (**B**) and 100 nM ISO (**C**). The bar plots show the differences between control, corrected and MFS CMs for RR interval (**D**), SDNN (**E**), SDSD (**F**) and coefficient of variance (**G**). Corrected CMs (n = 9, black), MFS CMs (n = 8, red). Poincaré plot from three representative replicates of the control (blue), corrected (black) and MFS CMs (red) (**H**). The three clusters of MFS are magnified in the right corner of figure (**H**). The level of significance is indicated by asterisks: *p* values less than 0.001 are indicated with three asterisks and *p* values less than 0.01 indicated with two asterisks.

CMs (15–20 day old) are passaged onto the MEA and the baseline is measured after a recovery period of 5 days. Homogenously beating cultures were observed and used for subsequent analysis. The beat-to-beat interval, depicted by the RR interval, is automatically extracted from the raw data (Fig. 5A–C). The electric field potential (EFP) of the baseline recordings (Fig. 5A) shows that the RR interval is significantly shorter in MFS CMs than in control (*p* < 0.0001) and corrected CMs (*p* = 0.0036) (Fig. 5D). Moreover, the variation of the RR interval at baseline measured as SDNN shows a significant difference between MFS CMs and control (*p* < 0.0001) and between MFS CMs and corrected CMs (*p* = 0.0031) (Fig. 5E). The SDSD also differs significantly between MFS CMs and control (*p* < 0.0001) and between MFS CMs and corrected CMs (*p* = 0.0045) (Fig. 5F). The coefficient of variance, which compensates for the difference in beat rate, also shows significantly less variance of the MFS CMs at baseline compared to control-CMs (*p* = 0.0007) and the corrected CMs (*p* = 0.0014) (Fig. 5G). The poincaré plot from three representative replicates per cell line shows that the RR intervals of MFS CMs cluster tightly together, while both control and corrected CMs show greater variations (Fig. 5H). This indicates that the MFS CMs show less beat-to-beat variability at baseline in comparison to corrected and control.

**Figure 6:**
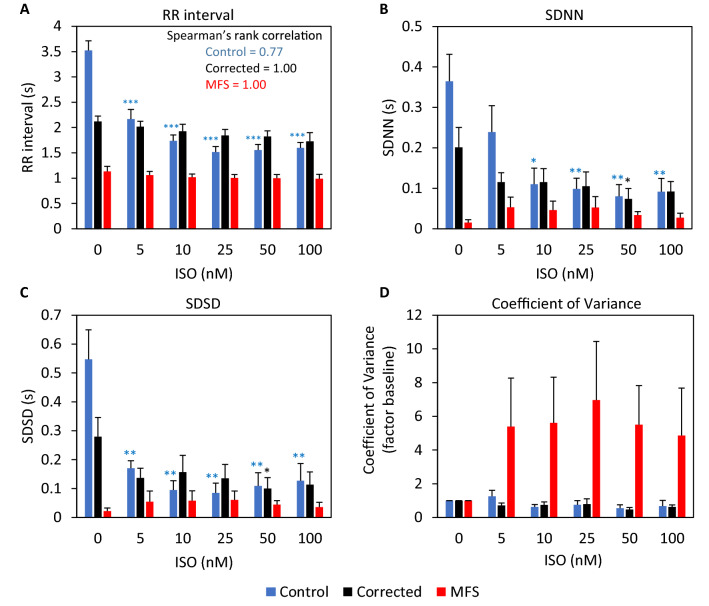
MEA measurements of control, corrected and MFS CMs after treatment with serial isoproterenol (ISO) concentrations of 5, 10, 25, 50 and 100 nM. Bar plots show differences between the three lines at each ISO concentration for RR interval (**A**), SDNN (**B**), SDSD (**C**) and the coefficient of variance presented as factor of the baseline (**D**). The Spearman’s rank correlation is indicated in panel A. Corrected CMs (n = 6, black), MFS CMs (n = 7, red), H9 control (n = 5, blue).

The effect of serial ISO treatment on CMs is measured using MEA. The EFP signals show that the control CMs beat significantly faster in response to higher concentrations (10 nM and 100 nM) of ISO (Fig. 5B,C). The RR interval of control CMs decreases with a higher concentration of ISO (Spearman’s rank correlation = 0.77) but a minimal and non-significant effect of ISO on the RR interval is observed for corrected and MFS CMs (Fig. 6A). Although both have a perfect correlation between the RR interval and the ISO concentration as indicated by Spearman’s rank correlation of 1. The beat-to-beat variation, as indicated by the SDNN (Fig. 6B) is significantly decreased for control CMs as a result of ISO treatment but no significant difference is observed for MFS CMs. For corrected CMs only a significant decrease is observed at 50 nM ISO treatment. The SDSD (Fig. 6C) is significantly decreased for the control at all the ISO concentrations and for corrected CMs only at 50 nM ISO treatment. While no significant effect for SDNN and SDSD is obtained for the MFS CMs, both parameters seem to increase in variation while the opposite is observed for both control and corrected CMs in response to ISO stress. Figure 6D shows the coefficient of variance relative to the baseline. This figure shows that the variation of the beat-to-beat interval increases in MFS CMs in response to ISO, however not significantly. The variance of the RR interval of both the control and corrected CMs remains similar after exposure to increased ISO concentrations when compared to baseline conditions.

### Chronic isoproterenol treatment reveals structural defects in ECM of MFS cell culture

**Figure 7:**
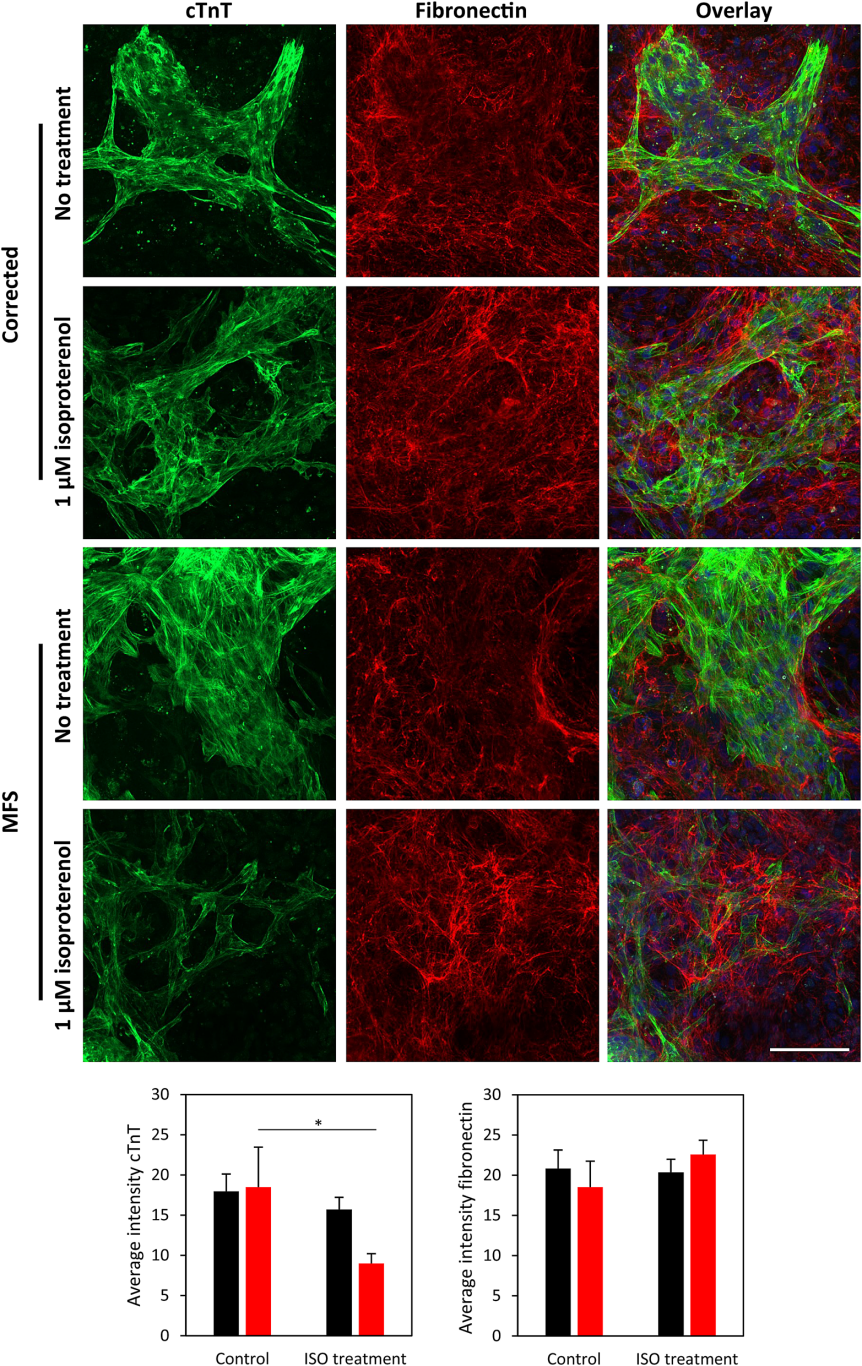
Cell-deposition of fibronectin. Chronic isoproterenol (ISO) treatment (1 µM) for 7 days for both MFS and corrected cell cultures is compared with no treatment using immunohistochemistry staining for cTnT and fibronectin, nuclei visualised with HOECHST. Bar indicates 100 µm. Bar graphs show average fluorescent intensity of cTnT and fibronectin for corrected (n = 3, black) and MFS (n = 3, red) with ISO treatment and no treatment. The level of significance is indicated by asterisks: *p* values less than 0.05 are indicated with one asterisk.

In order to test whether the fibrillin defect influences the CM culture morphology, the global architecture of fibrillary fibronectin deposited in the culture is visualized in 24-day-old cultures. To increase stress on the CMs, a treatment with 1 µM ISO was performed for 7 days, starting in cultures of 17 days old. The cell cultures present a higher abundance of fibronectin in areas of CMs, indicated by the cTnT staining, after ISO treatment compared to untreated controls (Fig. 7). In the untreated controls there is relative less fibronectin deposition in the areas of the CMs. Quantification of the average intensity of cTnT shows a significant reduction in ISO-treated versus non-treated MFS cell culture (Fig. 7 and suppl. Figure 3). A trend for increased fibronectin deposition is observed after ISO treatment for MFS CMs, although not significant. No significant reduction for cTnT nor fibronectin deposition is observed for corrected cell culture.

### Stretching of cell cultures leads to diminished level of MFS cardiomyocytes

**Figure 8:**
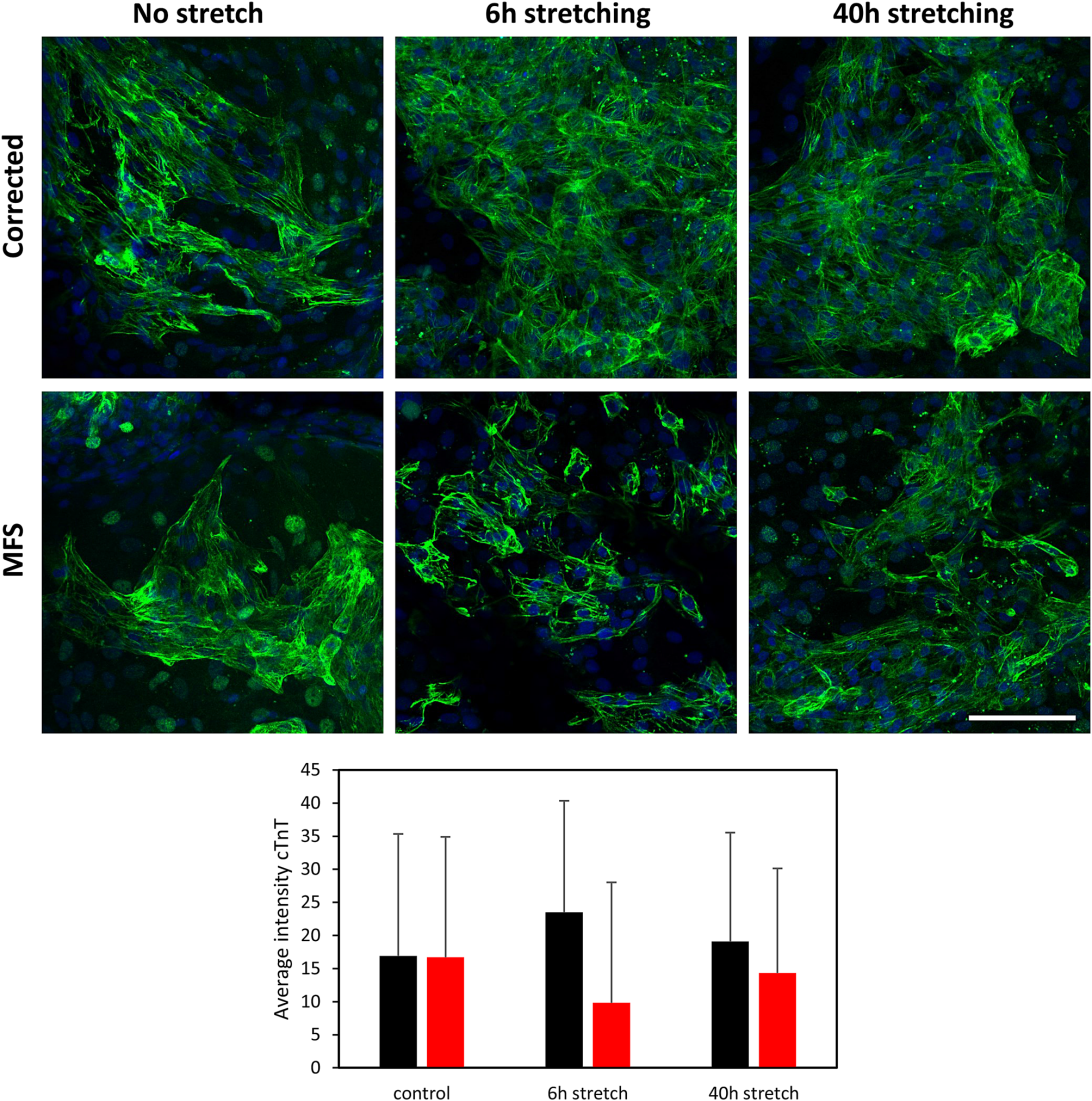
Stretching of in vitro cells to analyse structural integrity of the MFS CMs. Fluorescent images of immunohistochemistry staining for cTnT (green) after 6 h and 40 h of stretching using Flexcell and unstretched control (on membrane) for both MFS and corrected CMs. Bar plot shows the average intensity of cTnT in corrected (black) and MFS (red) CMs and includes the standard deviation. Bar = 100 µm.

Another set of experiments aim to create a dynamic strain on the CMs with a cyclic strain frequency to mimic the strain experienced by CMs in the myocardium using a Flexcell tension system on 25-day old-CMs. After 6 h and 40 h of stretching, cell cultures are stained for cardiac marker cTnT. MFS CMs appear less abundant after stretching and the layer of cells that are left appears impaired as indicated by more individual cTnT-positive cells (Fig. 8). The stress caused by stretching has no impact on the structural integrity of the CMs in the corrected cell culture.

### Substrate stiffness has no influence on beating rate of MFS cardiomyocytes

**Figure 9:**
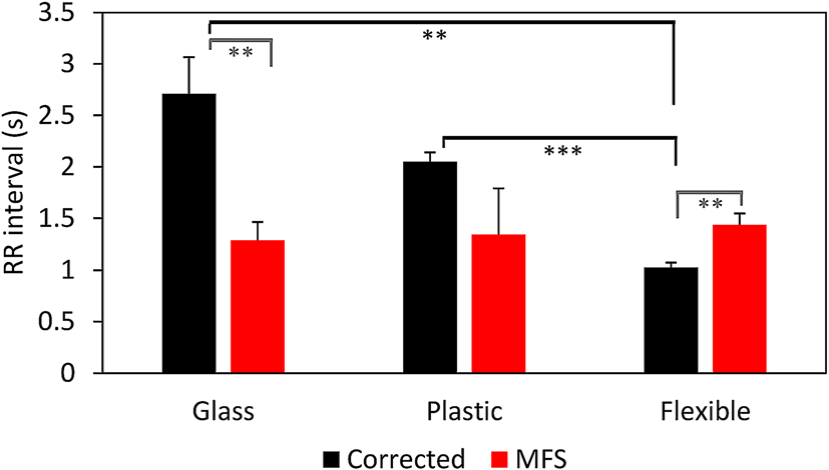
RR interval of corrected (black) and MFS (red) CMs measured on different substrate stiffnesses: glass (≈10 GPa) n = 8, plastic (≈0.1 GPa) n = 3 and on a flexible Flexcell membrane (≈150 kPa) n = 6. Error bars represent the standard error in the bar graphs. The level of significance is indicated by asterisks: *p* values less than 0.001 are indicated with three asterisks and *p* values less than 0.01 indicated with two asterisks.

Different substrate stiffness are used in this study, for MEA measurements glass (≈ 10 GPa), for AFM plastic (≈ 0.1 GPa) and for Flexcell a flexible membrane (≈ 150 kPa). The beat rate of CMs is analyzed to determine if different substrate stiffness has an influence on the functional behavior of the CMs. Significant differences in beat rate between corrected and MFS CMs are observed in case of flexible membrane (*p* = 0.006) and glass (*p* = 0.0036) (Fig. 9). When comparing the effect of the substrate on the beat rate of corrected CMs, significant difference between glass and flexible (*p* = 0.0022) and between plastic and flexible membrane (*p* < 0.0001) are observed. No significant differences in beat rate between the substrates are present for MFS CMs.

## Discussion

In literature there is accumulating evidence of MFS related cardiomyopathy. De Backer et al. reported primary impairment of the left ventricle unrelated to valvular disease^31^. Hetzer et al. described primary cardiomyopathy in 3% and secondary cardiomyopathy in 8% of MFS patients, which is more common than in the general population^32^. *FBN1* appears to be an effector of MFS cardiomyopathy, however these studies also warrant the necessity for a better understanding of the mechanisms responsible. Furthermore, another study demonstrates the high prevalence of ventricular arrhythmia in 48% of MFS patients^17^.

Data obtained in humans have been corroborated in several mouse models^13,33–35^. These findings also provide evidence for intrinsic myocardial dysfunction. Several mouse models for MFS have been established which resulted in increased knowledge about pathogenic pathways involved in MFS. However, there are important differences between mice and humans both genetically and physiologically, especially concerning the cardiovascular system. The hiPSC technology provides the opportunity to obtain difficult to access cell types such as CMs in a patient-specific manner, recapitulating the disease but missing some of the in vivo complexity^36^. In vitro derived CMs from hiPSCs have led to an increased understanding of various cardiomyopathies, including structural cardiomyopathies such as hypertrophic cardiomyopathy and other cardiomyopathies such as arrhythmogenic cardiomyopathy^37^.

Several hiPSC derived cell models for MFS have been established so far, modelling different aspects of the disease. The skeletogenic phenotype of MFS including osteogenic differentiation has been modeled in vitro^38^ and cell models for the vascular involvement in MFS were established by differentiating hiPSCs to vascular smooth muscle cells^25,39^. The skeletogenic model for MFS revealed impaired osteogenic differentiation as a result of increased TGF-β activation, and up-regulation of ECM markers *PAI-1* and collagen was observed^38^. In the vascular smooth muscle model for MFS, a reduced contractile phenotype was observed in response to carbachol^25,39^. The vascular smooth muscle model also showed an increase in *PAI-1* on mRNA level as a consequence of increased TGF-β signaling. Furthermore, the model showed specific differences in *TIMP* and *MMP* expression. Another in vitro study revealed that cultured fibroblast from skin biopsies have lower collagen and elastin gene expression in MFS compared to healthy volunteers^40^.

In this current study, we report the first, to the best of our knowledge, in vitro model for the MFS myocardium by differentiating hiPSCs to CMs. Functional experiments compared CM cell cultures derived from hiPSCs carrying a pathogenic variant in *FBN1* with CM cell cultures derived from the isogenic hiPSCs, where the pathogenic variant is CRISPR-Cas9 corrected. Corrected hiPSCs and MFS hiPSCs have comparable growth characteristics, and can be cultured in a similar manner (Fig. 1A,B). Based on the expression of the pluripotency markers OCT4 and SOX2, we can conclude that the pluripotency of the stem cells with the pathogenic variant in *FBN1* is unaffected (Fig. 1C,D), which is also described by Granata et al*.*^25^.

Also, *FBN1* did not affect the cardiac differentiation potential in this study and contraction of both corrected and MFS CMs is observed after 8–12 days after the start of differentiation. To assess cardiac maturation a molecular signature based on *TNNI* isoform expression of *TNNI1* (ssTnI, fetal) and *TNNI3* (cTnI, adult) was evaluated^41^. The maturation of MFS CMs seems to be hampered initially at day 15 when compared to corrected CMs, although not significantly (*p* = 0.074) (Fig. 1I). At day 24, the maturation status of the MFS CMs increased to a similar level as the corrected CMs. Similar gene expression of the cardiac specific gene *TNNT2* in the 24-days-old cultures (Fig. 1J) indicate a similar culture composition of CMs. Maturation of CMs is linked to their extracellular environment. The initial lack of maturation in MFS CMs could result from a dysfunctional matrix leading to decreased β1 integrin receptor activation and decreased focal adhesion kinase activity^42^. The herein presented cardiac in vitro model is composed of predominantly CMs which are growing in a contracting layer on top of a basal layer. Gene expression analysis reveals that both *TIMP1* and *KLF4* increase significantly at day 24 compared to day 15 in corrected CMs (Suppl. Figure 2). *KLF4*, *PAI-1* and *FBN1* expression significantly increases at day 24 compared to day 15 in MFS CMs. The morphology and characteristics of this CM cell culture system corresponds to previous described cardiac differentiations from mono-layer hiPSCs, as was first described by Lian and coworkers in 2012^43^.

The recovery and attachment after passaging is adequate in the corrected CMs (Fig. 1G). Normal fibrillin-1 and β1 integrin play an important role in the adhesion of CMs^44^. The fibrillin-1 deposition in the matrix of corrected CMs is normal (Fig. 2). AFM measurements confirmed that the matrix was stiffer than the CMs and indicate that the matrix is well developed in the corrected CMs. However, the recovery and attachment of the passaged MFS CMs is reduced compared to corrected CMs (Fig. 1H). The *FBN1* mutation in this model recapitulates an abnormal matrix as becomes clear from the fibrillin-1 deposition (Fig. 2). Measurements with AFM further elucidate a lesser developed matrix in the MFS cardiac model compared to the corrected model (Fig. 3). The perturbed matrix in the MFS cardiac model provides less support to the cell culture and could explain why the MFS CMs have more difficulty attaching. Collagen bundles are formed in the MFS cell culture as a potential response to the impaired fibrillin-1 deposition (Suppl. Figure 4), which may further impact the behavior of MFS CMs.

CMs in this in vitro cardiac model contract spontaneously. However, the MFS CMs show a reduced contraction amplitude compared to corrected CMs in the video analysis (Fig. 4). This finding is consistent with previous observations in smooth muscle cells of MFS^25^. AFM measurements reveal that the MFS CMs are stiffer in comparison to the corrected (Fig. 3), this could explain their reduced contraction amplitude.

A higher spontaneous beating rate in MFS CMs was observed with MEA measurements when compared to corrected CMs (Fig. 5). This divergence could be explained by the differences in the ECM composition as indicated by AFM measurements, since the ECM influences the beating rate of in vitro CMs^45^. The effect of the substrates used in this study with different stiffness; glass (MEA, ≈ 10 GPa), plastic (AFM, ≈ 0.1 GPa) and flexible (Flexcell, ≈ 150 kPa), on the beating rate of CMs was further investigated. Corrected CMs were influenced by the substrate and have a significantly smaller RR interval when cultured on a flexible membrane compared to glass (Fig. 9). Previous studies also concluded that the optimal stiffness of the substrates (between 10 and 30 kPa) for CMs is important for their normal phenotype and that CMs beat faster when cultured on substrates with a lower stiffness^46,47^. However, MFS CMs did not show a different beating rate at the different substrates. The abnormal fibrillin-1 in MFS cultures is thought to disconnect the CMs from their extracellular environment.

Interestingly, the variation in the beating rate as measured with MEA was high in the corrected CMs, while the CMs from MFS were beating with little variation (Fig. 5). This arrhythmogenic behavior of the corrected CMs was replicated with CMs derived from the hESC H9 control line. MEA measurements on fibronectin coated glass substrate have been reported to cause more beating variation, compared to more ideal culture systems such as hydrogels^48^, that more closely resemble the in vivo extracellular environment. Since the ECM is assumed to be abnormal in MFS, it could be suggested that the MFS CMs receive less mechanical feedback signals from the environment, explaining the small amount of variation in beat rate^49^. Other studies describe that CMs derived from hiPSCs and hESCs exhibit intrinsic beat rate variability resembling pacemaker cells in the adult heart without the autonomic nerve system being present in the in vitro system^50,51^. This suggest an intrinsic cardiac regulatory mechanism which is thought to be influenced by dynamic structural, biochemical and intracellular changes. For instance, single CMs have a higher beat-to-beat rate variability compared to CMs in embryoid body configuration^52^.

ISO mimics increased sympathetic nerve activity and stress. The acute response to ISO did not show a significant decrease in the RR interval for the corrected and MFS CMs, but showed a perfect correlation between increasing ISO concentration and decreasing RR interval (Fig. 6). The variation of the RR interval decreased in the corrected CMs and increased in the MFS CMs. The impact of chronic stress, which mimics advanced heart failure by means of chronic ISO treatment was more extensive on the MFS CMs (Fig. 7). Immunofluorescent staining showed that the cTnT was significantly reduced after ISO treatment in the MFS cell culture but maintained in the corrected cell culture. The overall fibronectin deposition remained similar after ISO treatment. However, at the locations of diminished cTnT signal a trend for increased fibronectin deposition was observed. We postulate that the CMs in these areas experience the most severe stress. Fibronectin co-localizes with fibrillin-1 and is essential for the formation of microfibrils^53^. β1 integrin is important in the connection between the matrix and CMs and is also implicated in the response to beta adrenergic stimulation as an important transmitter of this stress^54^. The deposition of fibronectin is important for a healthy response to the increased stress and thus a demand for more structural support of the ECM as is observed in the corrected cell-culture. The lack of structural support in the MFS cell culture could lead to the more disrupted appearance of the MFS CMs observed after the ISO treatment or even CM cell death^54^.

Rouf and colleagues discovered that in the *Fbn1*^*c1039G/*+^ mouse model for MFS, the unstressed heart did not reveal cardiac structural or functional abnormalities^34^. Only when increased hemodynamic load was posed on the hearts of these mice by means of transverse aortic constriction, ventricular dilatation and dysfunction was observed. This coincides with the Flexcell results. Flexcell revealed in the in vitro cardiac model that the MFS CMs were less abundantly present after stretching and that the monolayer seemed to be impaired after stretching (Fig. 8). The MFS CMs seem more prone to this simulated hemodynamic stress, likely caused by their weaker ECM unable to handle the mechanical stimuli, unlike the corrected CMs. This confirms that fibrillin-1 is essential for a normal response to increased mechanical load.

Taken together, the deposition of fibrillin-1 in the ECM was disrupted in the described in vitro cardiac model for MFS and is thought to be the driving force for the observed abnormalities. The findings in this study coincide with the conclusions made by Cook and colleagues that fibrillin-1, present in the ECM, is important to adapt the CMs to increased stress^14^. This implies that CMs with dysfunctional fibrillin-1 will not adapt as well to stress compared to corrected CMs. Our findings suggest that the MFS CMs are more disconnected from their ECM environment, possibly via decreased β1 integrin levels. This would impair correct sensing of increased stress from the ECM to the CMs and vice versa.

The observed abnormalities in our in vitro cardiac model for MFS could have a detrimental effect on functioning of the heart as a whole. A subset of patients develop MFS related cardiomyopathy, but the mechanism is still unclear. MFS CMs in our model prove to be much more vulnerable to stress in comparison to corrected CMs. This poses the idea that MFS related cardiomyopathy is stress-induced, possibly caused by impaired and less supportive matrix. However, it should be considered that this in vitro cardiac model may oversimplify the in vivo situation in MFS and does not account for all the complexity of the syndrome. The impairment of the MFS CMs could occur in another, much slower fashion in vivo. The stress posed on the in vitro CMs by means of ISO and Flexcell could be an exaggeration of the stress actually present in a human being, but was used to evoke a potential response. This proof of concept study (one mutation) reveals distinct differences in the MFS CMs compared to the isogenic control by the use of functional assessments. Moreover, the established model could form the fundamental support for future research. Exploration of the observed abnormal behavior of MFS CMS is warranted and should address the behavior of CMs on a cell level in terms of sarcomere structure and deformation.

The complex pathologic mechanisms that drive MFS remain unclear, and in this study the highly variable phenotype caused by over 3000 known pathogenic variants in *FBN1* gene and its impact on the functional characteristics of the CMs is not addressed. This proof of concept study provides evidence that CMs show abnormal behavior in the context of MFS using different functional characterization methods. We postulate that impaired mechanosensing via β1 integrin could explain the observed results, but this mechanism should be further elucidated. However, it remains to be answered if the defects in the ECM are solely driving the observed functional differences in the CMs, or if the CMs contribute as well. This warrants further investigation into MFS cardiomyopathy. The described in vitro cardiac model for MFS demonstrates phenotypic differences in the MFS CMs, providing an interesting platform to further study disease mechanisms and for the assessment of new therapies for MFS.

## Supplementary information

Supplementary Informations.

Supplementary Figure 1.

Supplementary Figure 2.

Supplementary Figure 3.

Supplementary Figure 4.

Supplementary Video 1.

Supplementary Video 2.
