# Supplementary material for: Effects of fibrillin mutations on the behavior of heart muscle cells in Marfan syndrome
Source: Sci Rep. 2020 Oct 7;10:16756. doi: 10.1038/s41598-020-73802-w (PMC7542175; doi:10.1038/s41598-020-73802-w)
Supplement: Supplementary file 1 — Supplementary Informations. [file 41598_2020_73802_MOESM1_ESM.docx]

**Effects of fibrillin mutations on the behavior of heart muscle cells in Marfan syndrome**

Jeffrey Aalders^a^, Laurens Léger^a^, Louis Van der Meeren^b^, Natasja Van den Vreken^a^, Andre G. Skirtach^b^, Sanjay Sinha^c^, Julie De Backer^d^ and Jolanda van Hengel^a^

^a^ Medical Cell Biology research group, Department of Human Structure and Repair, Faculty of Medicine and Health Sciences, Ghent University, Ghent, Belgium

^b^ Department of Biotechnology, Faculty of Bioscience Engineering, Ghent University, Ghent, Belgium

^c^ Wellcome-MRC Cambridge Stem Cell Institute, University of Cambridge, Cambridge, United Kingdom

^d^ Center for Medical Genetics, Ghent University Hospital, Ghent, Belgium

**Corresponding author:**

Jolanda van Hengel, Ph.D., Medical Cell Biology research group, Department of Human Structure and Repair, Faculty of Medicine and Health Sciences, Ghent University, Corneel Heymanslaan 10, Building B, entrance 36, B-9000 Ghent, Belgium.

Tel: +32.9.33 24009

e-mail: Jolanda.vanhengel@ugent.be

ORCID iD: [0000-0003-0645-0435](http://orcid.org/0000-0003-0645-0435)

Supplementary data

| **Antibody** | **Dilution** | **Company (Cat no.)** | **Remarks** |
| --- | --- | --- | --- |
| Mouse-anti-OCT4 | 1:700 | Santa Cruz (Sc-5279) | Stem cells |
| Rabbit-anti-SOX2 | 1:600 | Life Technologies (PA1-094) | Stem cells |
| mouse monoclonal anti-cTnT | 1:250 | Life Technologies (MA5-12960) | Cardiomyocytes |
| rabbit polyclonal anti-fibronectin | 1:100 | Life Technologies (PA5-29578) | Cardiomyocytes |
| mouse monoclonal anti-fibrillin-1 | 1:100 | Kindly provided by Prof. Dr. Lynn Sakai | Cardiomyocytes |
| Goat-anti-Mouse IgG Dylight 488 | 1:1000 | Life Technologies (35503) | Secondary antibody |
| Goat-anti-Rabbit IgG Dylight 594 | 1:500 | Life Technologies (35561) | Secondary antibody |

Supplementary table 1: primary and secondary antibodies are listed that are used for immunofluorescent stainings of stem cells and cardiomyocytes.

| **Primer** | **Forward** | **Reverse** | **bp** | **Reference** |
| --- | --- | --- | --- | --- |
| *TNNT2* | TTCACCAAAGATCTGCTCCTCGCT | TTATTACTGGTGTGGAGTGGGTGTGG | 166 | ^1^ |
| *TNNI1* | TGGATGAGGAGCGATACGAC | TTGTGAGGTCGGAGACTTGG | 335 |  |
| *TNNI3* | CGATGCGGCTAGGGAACCTC | TGCAATTTTCTCGAGGCGGA | 126 |  |
| *TIMP1* | CCTTCTGCAATTCCGACCTC | GTATCCGCAGACACTCTCCA | 185 | ^2^ |
| *KLF4* | ATCTTTCTCCACGTTCGCGTCTG | AAGCACTGGGGGAAGTCGCTTC | 121 | ^3^ |
| *PAI-1* | CCGCCTCTTCCACAAATCAG | AATGTTGGTGAGGGCAGAGA | 202 | ^2^ |
| *FBN1* | ACCGTGCTTTTAGCGTCCTA | GGCAAATGGGGACAATACAC | 217 | ^4^ |
| *B2M* | TGCTGTCTCCATGTTTGATGTATCT | TCTCTGCTCCCCACCTCTAAGT | 86 |  |
| *RPL13A* | CCTGGAGGAGAAGAGGAAAGAGA | TTGAGGACCTCTGTGTATTTGTCAA | 126 |  |
| *GAPD* | TGCACCACCAACTGCTTAGC | GGCATGGACTGTGGTCATGAG | 87 | ^5^ |

Supplementary table 2: Primer sequences listed that are used for RT-qPCR experiment. Primers for RT-qPCR were used at a final concentration of 200 nM. Gene expression was normalized based on reference genes *Beta-2-Microglobulin* (*B2M*), *Ribosomal Protein L13a* (*RPL13A*) and *Glyceraldehyde-3-phosphate dehydrogenase* (*GAPD*) with GeNorm^5^.

Supplementary video 1: Representative movie of corrected cardiomyocytes. Movies were taken at 30 frames per second.

Supplementary video 2: Representative movie of Marfan cardiomyocytes. Movies were taken at 30 frames per second.

Figure legends:

Suppl. Fig. 1: Immunofluorescent pictures from stem cell cultures of corrected and MFS. The hiPSC cultures show expression of pluripotency marker NANOG (red) and HOECHST (blue). Scalebar indicates 500 µm.

Suppl. Fig. 2: Normalized gene expression in corrected and MFS cell cultures at 15d (grey and red respectively) and 24d (black and dark-red respectively) for *FBN1*, *PAI-1*, *KLF4* and *TIMP1*. The graphs present the average of 3 independent cultures per condition including standard error bars. Gene expression was normalized based on reference genes *B2M*, *RPL13A* and *GAPD*. No significant differences between corrected and MFS were reached. The primer sequences are listed in supplementary table 2.

Suppl. Fig. 3: Additional images used for analysis of cell-deposition of fibronectin. Chronic isoproterenol (ISO) treatment (1 µM) for 7 days for both MFS and corrected cell cultures is compared with no treatment using immunohistochemistry staining for cTnT and fibronectin, nuclei visualised with HOECHST. Bar indicates 100 µm.

Suppl. Fig. 4: Fluorescent visualization of collagen fibers using picrosirius red staining. Cell cultures were stained at day 15 and day 24 after start of cardiac differentiation. 17 days old CMs from corrected and MFS were stressed with chronic isoproterenol (ISO) treatment (1 µM) for 7 days and compared to 24 days old cell cultures without treatment. Collagen fibers are present in the ECM of ISO stressed CMs of corrected but are limited in the MFS cell culture after ISO stress. Bar = 400 µm.

Supplementary materials:

**Picrosirius red staining** CMs were fixed for 20 min with 4% paraformaldehyde at RT. Coverslips were stained for 30 min at RT with 0.2% Sirius red F3B (Sigma-Aldrich, cat no. 365548) in a saturated solution of picric acid (Sigma-Aldrich, cat no. 80450). They were subsequently washed with distilled water and dehydrated in increasing series of ethanol (70%, 96%), isopropanol, xylene and mounted with mounting medium. Fluorescent images were taken to visualize the collagen stained fibers using EVOS FL Imaging System (Thermo Fisher Scientific, cat no. AMF4300)^6^.

1 Kattman, S. J. *et al.* Stage-specific optimization of activin/nodal and BMP signaling promotes cardiac differentiation of mouse and human pluripotent stem cell lines. *Cell Stem Cell* **8**, 228-240, doi:10.1016/j.stem.2010.12.008 (2011).

2 Granata, A. *et al.* An iPSC-derived vascular model of Marfan syndrome identifies key mediators of smooth muscle cell death. *Nat. Genet.* **49**, 97-109, doi:10.1038/ng.3723 (2017).

3 Moon, J. S. *et al.* Kruppel-like factor 4 (KLF4) activates the transcription of the gene for the platelet isoform of phosphofructokinase (PFKP) in breast cancer. *J. Biol. Chem.* **286**, 23808-23816, doi:10.1074/jbc.M111.236737 (2011).

4 Tojais, N. F. *et al.* Codependence of Bone Morphogenetic Protein Receptor 2 and Transforming Growth Factor-beta in Elastic Fiber Assembly and Its Perturbation in Pulmonary Arterial Hypertension. *Arterioscler. Thromb. Vasc. Biol.* **37**, 1559-1569, doi:10.1161/ATVBAHA.117.309696 (2017).

5 Vandesompele, J. *et al.* Accurate normalization of real-time quantitative RT-PCR data by geometric averaging of multiple internal control genes. *Genome Biol.* **3**, RESEARCH0034, doi:10.1186/gb-2002-3-7-research0034 (2002).

6 Vogel, B., Siebert, H., Hofmann, U. & Frantz, S. Determination of collagen content within picrosirius red stained paraffin-embedded tissue sections using fluorescence microscopy. *MethodsX* **2**, 124-134, doi:10.1016/j.mex.2015.02.007 (2015).
