# Supplementary figures and images for: Effects of fibrillin mutations on the behavior of heart muscle cells in Marfan syndrome
Source: Sci Rep. 2020 Oct 7;10:16756. doi: 10.1038/s41598-020-73802-w (PMC7542175; doi:10.1038/s41598-020-73802-w)

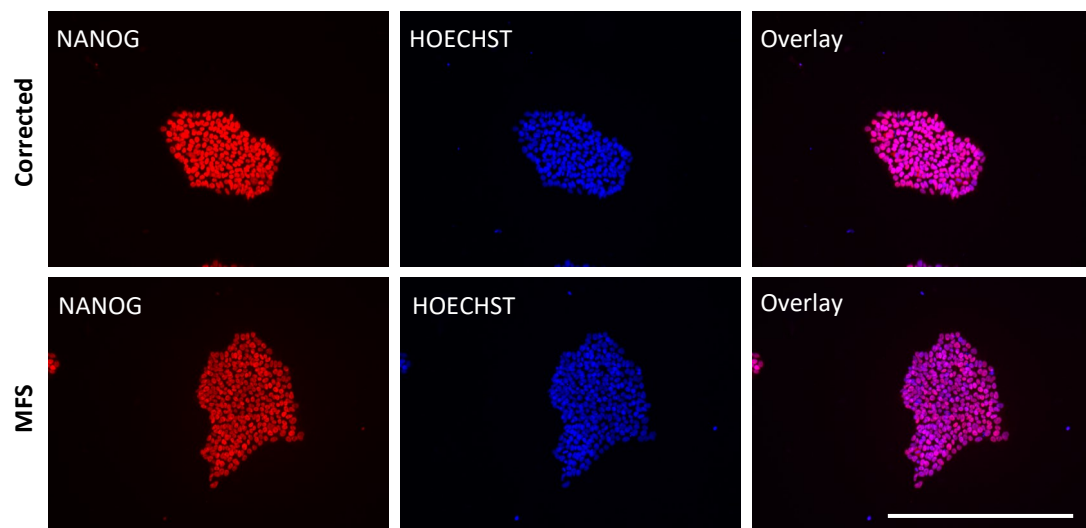

Supplement: Supplementary file 2 — Supplementary Figure 1. [file 41598_2020_73802_MOESM2_ESM.pdf]

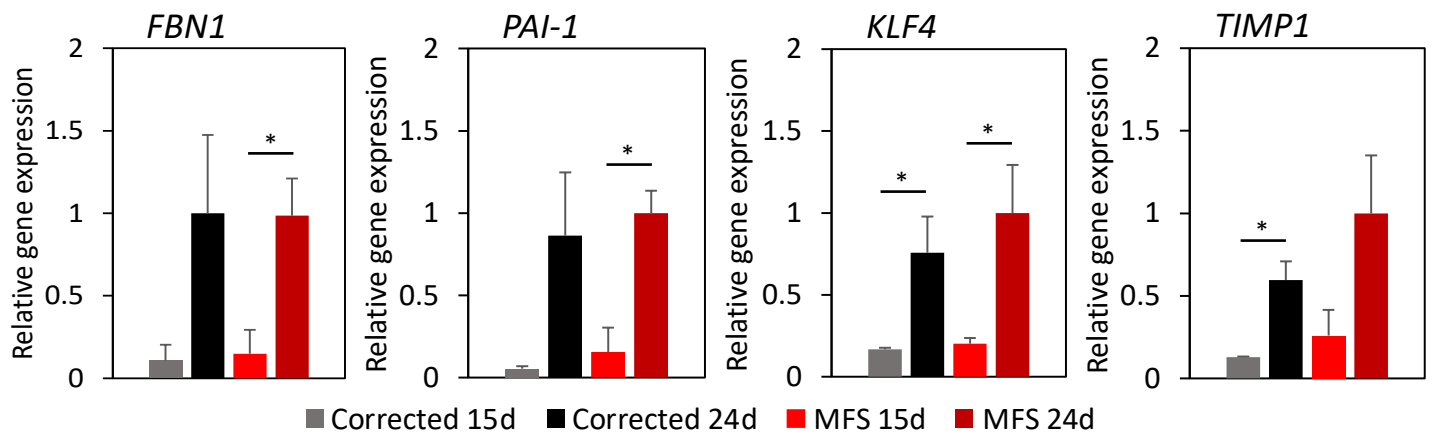

Supplement: Supplementary file 3 — Supplementary Figure 2. [file 41598_2020_73802_MOESM3_ESM.pdf]

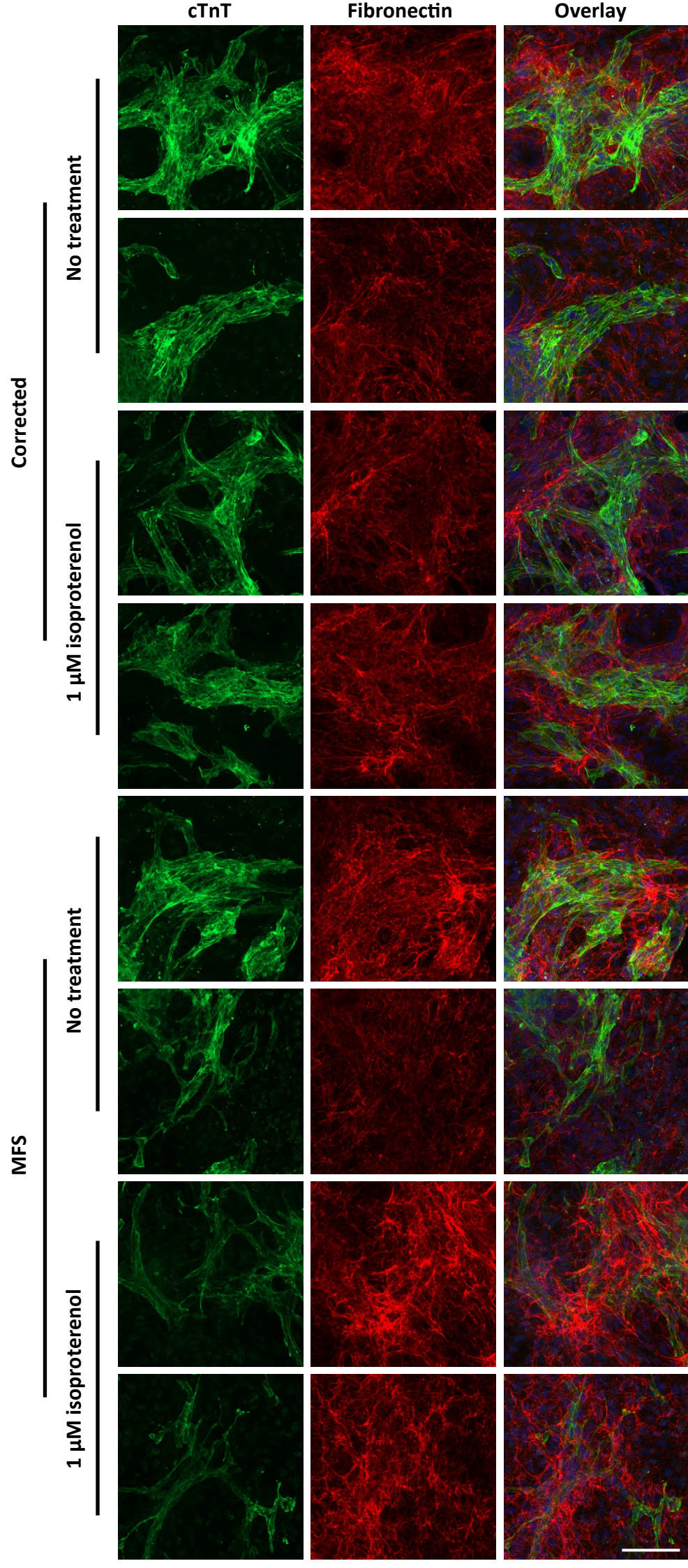

Supplement: Supplementary file 4 — Supplementary Figure 3. [file 41598_2020_73802_MOESM4_ESM.pdf]

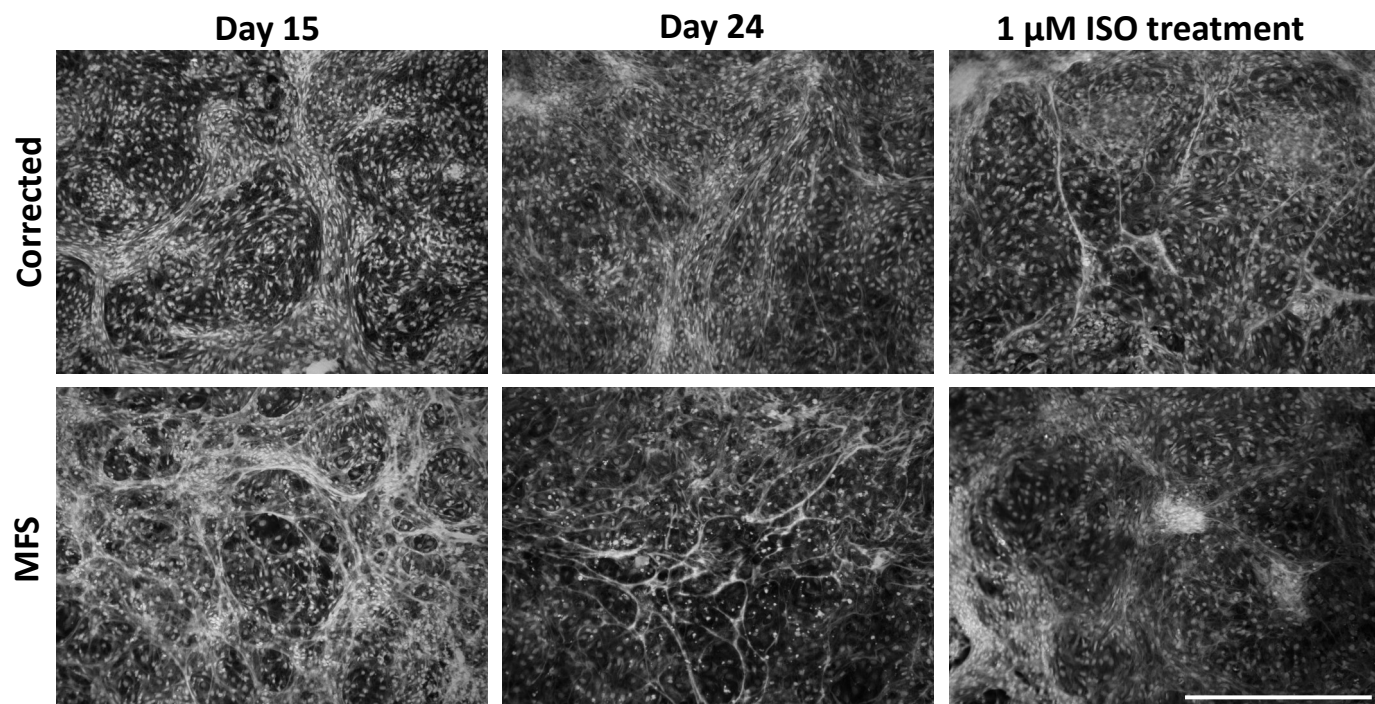

Supplement: Supplementary file 5 — Supplementary Figure 4. [file 41598_2020_73802_MOESM5_ESM.pdf]
